# Impact of improved recording of work-relatedness in primary care visits at occupational health services on sickness absences: study protocol for a randomised controlled trial

**DOI:** 10.1186/s13063-017-2076-3

**Published:** 2017-07-26

**Authors:** Salla Atkins, Ulla Ojajärvi, Nina Talola, Mervi Viljamaa, Jaakko Nevalainen, Jukka Uitti

**Affiliations:** 1 Faculty of Medicine and Life Sciences, University of Tampere, Tampere, Finland; 2 Pihlajalinna Työterveys, Tampere, Finland; 3 Faculty of Social Sciences, University of Tampere, Tampere, Finland; 4 Department of Public Health Sciences, Karolinska Institutet, Stockholm, Sweden

**Keywords:** Occupational health care, Sickness absence, Work ability, Cluster randomised trial, Electronic patient registers

## Abstract

**Background:**

Employment protects and fosters health. Occupational health services, particularly in Finland, have a central role in protecting employee health and preventing work ability problems. However, primary care within occupational health services is currently underused in informing preventive activities. This study was designed to assess whether the recording of work ability problems and improvement of follow-up of work-related primary care visits can reduce sickness absences and work disability pensions after 1 year.

**Methods/design:**

A pragmatic trial will be conducted using patient electronic registers and registers of the central pensions agency in Finland. Twenty-two occupational health centres will be randomised to intervention and control groups. Intervention units will receive training to improve recording of work ability illnesses in the primary care setting and improved follow-up procedures. The intervention impact will be assessed through examining rates of sickness absence across intervention and control clinics as well as before and after the intervention.

**Discussion:**

The trial will develop knowledge of the intervention potential of primary care for preventing work disability pensions and sickness absence. The use of routine patient registers and pensions registers to assess the outcomes of a randomised controlled trial will bring forward trial methodology, particularly when using register-based data. If successful, the intervention will improve the quality of occupational health care primary care and contribute to reducing work disability.

**Trial registration:**

ISRCTN Registry reference number ISRCTN45728263. Registered on 18 April 2016.

**Electronic supplementary material:**

The online version of this article (doi:10.1186/s13063-017-2076-3) contains supplementary material, which is available to authorized users.

## Background

There is a strong relationship between employment and health. Full-time employment protects and fosters health [1–3]. The beneficial effects of work on perceived health status and quality of life can be seen even after re-employment after a period of unemployment [4]. Because of the importance of work for both individual well-being and health, along with national productivity, it is important to ensure that individuals are healthy, maintain their work ability and are able to participate in the workforce. Maintaining work ability, however, is not a simple process. There are several identified risk factors for work disability, including work characteristics [5, 6]; unstable jobs and unemployment [7]; a number of chronic diseases [8]; sickness absence rates [9]; and some personal characteristics, such as socioeconomic characteristics [6], education level [10], gender [11] and age [10]. Most of these risk factors for work disability are related to workplace characteristics [12], and employers can influence these factors by leadership, organisational changes and working arrangements [13]. For effective action, the employer needs collaboration with occupational health services (OHS) that can provide information regarding the early signs of work disability. Collaboration with OHS can continue from early signs of work disability to managing ongoing work disability, such as by modifying work tasks or supporting return to work [14].

Work ability is a central issue in Finland because 7.5% of the working population retire early on disability pensions, which also lowers the average retirement age [5] and productivity. In Finland, OHS monitor and maintain employee work ability and through various activities to prevent work disability and other work-related health problems [15]. The wide mandate of OHS is possible because all employees are required to have preventive OHS by law, and approximately 80% of the working population also receive primary care services through OHS. Investments in OHS have resulted in benefits for organisations and their employees, such as reduction in sick leave [16] and increased profitability [16, 17]. In recent years, disability pension rates have also declined [18]. In Finland, OHS are an important provider of health care, parallel to the private and public sectors [15]. OHS are at a key position of fostering employees’ work ability because they see employees during both preventive and curative activities.

Though progress has been made in reducing work disability, more work is needed in this area, and OHS potential in this respect is underused. There is insufficient information about the extent, impact and actions taken, based on assessments of work ability or diagnoses’ work-relatedness during these visits. Over half of the causes of these visits are work-related (27% caused by work and 52% impair work ability) [19], but this information is rarely used in preventive activities because electronic records do not allow for precise collection and use of this information. Intervening in working conditions from the primary care setting could contribute to improving employee work ability and reducing disability [20].

The trial described in this article is a multi-centre, pragmatic, parallel-group, cluster randomised controlled trial using electronic health records and pension registries to improve the recording of work-relatedness and impact on work ability of primary care visits and ultimately the follow-up of these cases at OHS units in Finland. We follow the standardised Standard Protocol Items: Recommendations for Interventional Trials (SPIRIT) checklist in this report (Additional file 1).

### Aim 1

Our first aim in the present study is to assess the effect of an intervention for improving recording and follow-up of patient primary care visits in occupational health care on medium-length sickness absence. Our hypothesis is that improved recording of work-relatedness and/or effects on work ability during primary care visits and closer follow-up and initiation of actions involving the patient and employer at the workplace will reduce medium-length (4–9 days) sickness absences.

### Aim 2

Our second aim in the present study is to assess the effect of an intervention to improve the recording and follow-up of primary care visits to OHS on work disability pensions as recorded in the central pensions register and long-term sickness absence (9–60 days) as recorded in electronic patient registers. We will also investigate the impact of the intervention on short-term (1–3 days) sickness absences. Our hypothesis is that closer patient follow-up will enable the occupational health (OH) and employer teams to address work ability problems earlier and thus reduce sickness absences and disability pension rates.

### Research question

Does an intervention to improve recording and follow-up of primary care visits’ work-relatedness and their impact on work ability reduce medium-length sickness absences (4–9 days) more than no intervention when measured after 1 year?

## Methods/design

### Trial design

The trial is a multi-centre, parallel-group, pragmatic superiority, cluster randomised controlled trial of 1 year in duration. Each OH unit represents one cluster. Because the intervention is provided to doctors responsible for multiple patients and multiple employer organisations, a cluster design was chosen to decrease contamination bias. The study design was developed in accordance with the SPIRIT guidelines checklist.

### Setting

The study was developed together with Pihlajalinna Työterveys, an OHS provider that is part of a private consortium. At the time of trial initiation, the provider has 22 OH units in different cities and towns across Finland, which together had approximately 67,000 employees on their client lists at the end of 2015. In Finland, approximately 91% of all employees are within the remit of OHS [21], approximately 86% of whom have company-provided primary care services, with the rest receiving legislative preventive services.

### Participants and recruitment

Each OH unit within Pihlajalinna Työterveys had between 500 and 9000 employees on their register. In the study, each cluster is considered an independent unit. All OH units in the consortium with full-time OH staff were selected for the study. Two centres within the same town as a larger centre with part-time OH staff were left out of the study. The intervention training was considered routine staff training. Employees on the register of the OHS in 2015–2017 between the ages of 18 and 65 years will be included in the analysis. The cohort will be open, and employees are eligible if they join the OH register at any time during the study period. An employee visiting any of the 22 OH units were considered eligible for analysis.

### Randomisation and allocation

To allocate OH units into intervention and control groups, a randomisation process using dynamic allocation (specifically minimisation) was used. This method was first presented by Taves [22] and later by Pocock and Simon [23]. The use of minimisation has increased in recent years [24], and it has been suggested as the ‘platinum standard’ of trials [25] because it allows for balance between treatment and control arms. In this trial, we considered the following as potential confounders and included them in the minimisation: (1) the client volume of the OHS separated into three enterprises—small, medium and large, (2) the main sector of the OHS clients (industry vs others) and (3) whether one of the OHS unit’s enterprise clients was a major paper factory or not (the paper factory had instituted large-scale OH interventions in recent years).

We began the randomisation with simple randomisation conducted by the team statistician (NT). We allocated the first four units through simple randomisation (1:1) using the random number generator in the R program (R Foundation for Statistical Computing, Vienna, Austria). For the remaining 18 units, we used minimisation to allocate each OHS unit to either the treatment group or the control group to minimise the total imbalance for potential confounders, continuing with a 1:1 ratio. Because the intervention was embedded in routine practice, allocation was concealed from participants but not from providers or investigators. The concern of lack of blinding during the minimisation process did not apply in this case, owing to the cluster-based design and the fact that both the intervention and control clusters are part of Pihlajalinna Työterveys.

### Assessment points

**Fig. 1:**
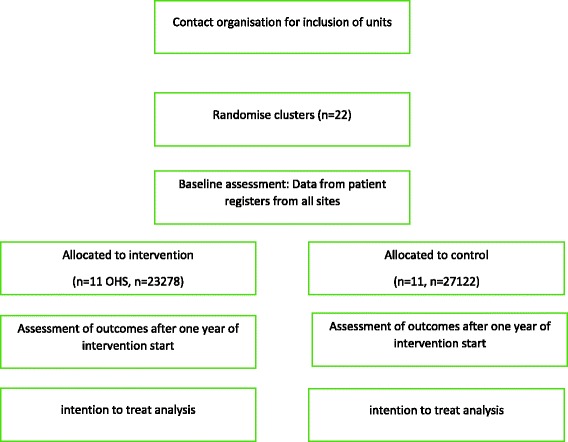
Trial flow diagram. *OHS* Occupation health services

**Fig. 2:**
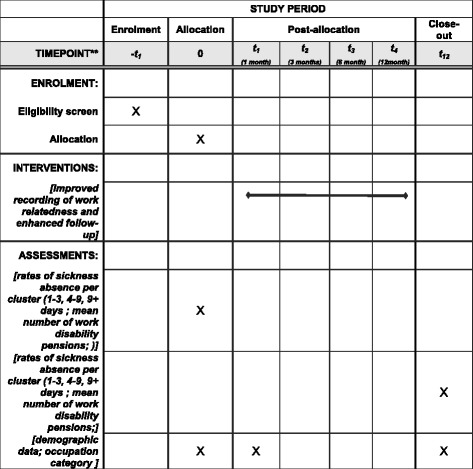
SPIRIT figure: Summarizes the allocation, interventions, and outcomes of the study

Baseline information will be assessed for 1 year preceding the intervention, from 1 May 2015 to 1 May 2016. Approximately 72,000 spells of sickness absence were registered among 25,000 clients at Pihlajalinna Työterveys in 2015. For the primary outcome (mean sickness absence of medium length after the intervention compared with baseline), assessment will be conducted by NT after 1 year of the intervention, using data up to 1 May 2017 (*see* Fig. 1 for trial flow and Fig. 2 for SPIRIT diagram). For mean disability pensions per centre, the time will be increased to 31 December 2017, with subsequent analysis hoped for after more funding is received.

### Intervention

All units were informed of the ongoing research and its aims before the start of training. In the intervention group, all doctors and nurses or other OH professionals who are part of intervention units took part in training provided by Pihlajalinna Työterveys. The intervention training started on 1 May 2016. The training lasted approximately 1 h. During the training, the principle of the intervention was explained, and all participants received written instructions on the recording procedure and intervention processes. When physicians or nurses were absent, a follow-up visit or phone call was made to impart the intervention information. Each physician and nurse responsible for a company’s OHS took responsibility for their companies’ employees’ follow-up. The implementation of the intervention was followed by the Pihlajalinna Työterveys trainer, who followed up with nurses and physicians and invited them to personal discussions on the process as necessary.

**Fig. 3:**
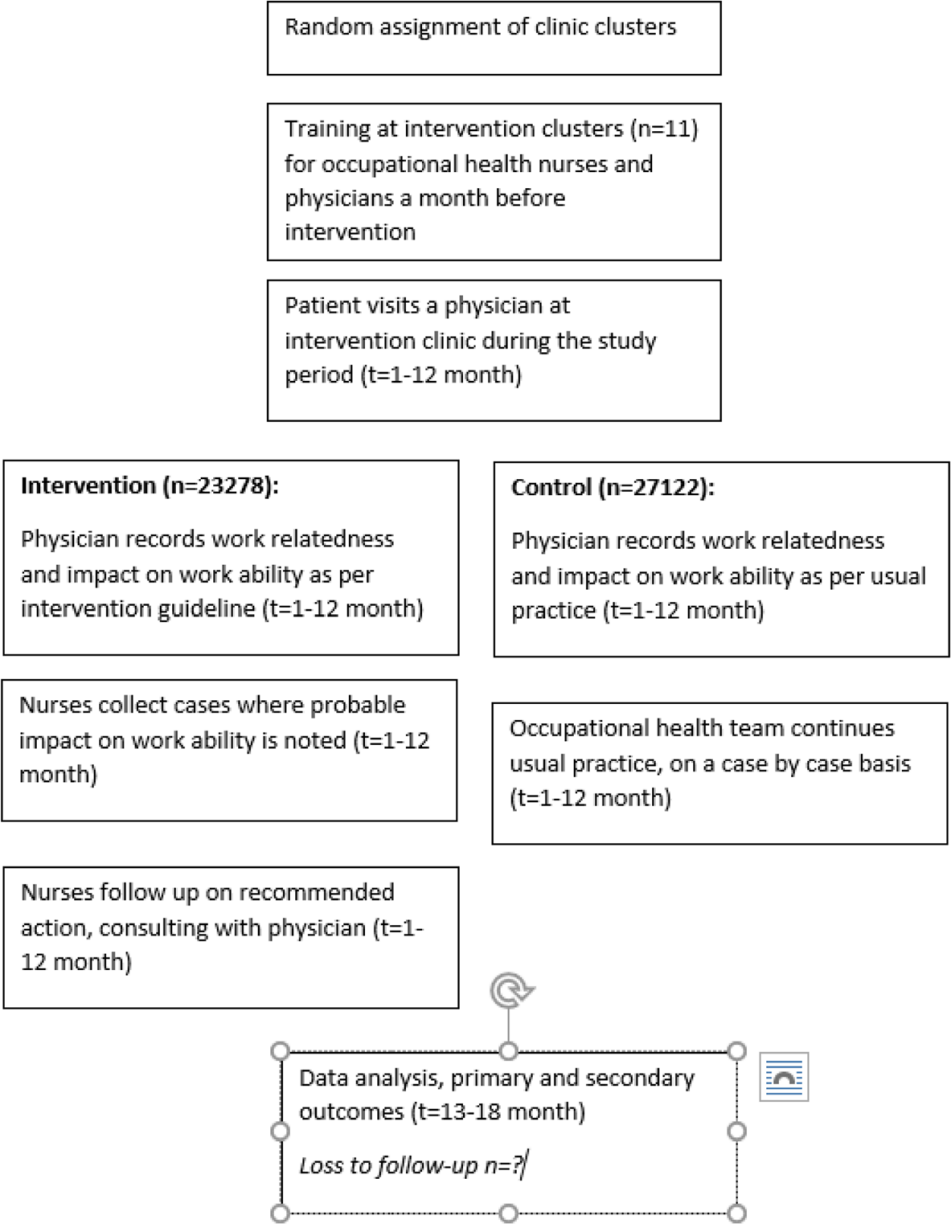
Participant flow diagram

Figure 3 details the intervention and participant flow. The intervention was designed to first estimate the relationship of the main or additional diagnosis or the visit reason to work. During each primary care visit, the attending physician would assess firstly whether the diagnosis was related to work. Secondly, the client’s risk of applying for a work disability pension was estimated according to four categories: (1) no risk of work disability pension, (2) less than 50% chance of work disability pension in the near future (2–5 years), (3) more than 50% chance of work disability pension in the near future (2–5 years) and (4) immediate risk of work disability pension. This assessment is entered in Pihlajalinna Työterveys’s electronic patient register. Once weekly, all patients are recalled on the electronic system for whom a record of work-relatedness was made. Each case is then assigned to the OH nurse responsible for the patient’s employer relations. The responsible OH nurse then initiates follow-up visits as appropriate. These can include worksite visits; tripartite negotiations between the OH physician, employee and employer; occupational physiotherapy for the employee; occupational psychologists’ assessments; or different kinds of rehabilitation processes at an individual level. When a client is deemed as at greater than 50% risk of work disability, a separate rehabilitation plan will be completed with the client present. This plan will include details on the client’s health status, planned actions for rehabilitation, and follow-up by the OH professionals.

The control group did not receive additional training on recording work-relatedness. It is possible that Control group physicians might hear of the intervention and might pay more attention to assessing potential threats to work ability during the intervention. However, no central information is provided regarding the intervention in the consortium, and no formalised information is provided to control units, which may reduce confounding. After the end of the intervention, control units will be given a chance to participate in training and implement the intervention if the results of the intervention were positive. Compliance is checked via regular contacts by the intervention trainer with the OH units, enquiring about implementation and challenges.

### Outcome measures

#### Primary outcome

The primary outcome is reduction from baseline of the mean number of medium term (4–9 days) sickness absences from the workplace per intervention and control centre after 1 year of follow-up as measured by OHS patient records. We chose medium-length sickness absences as the primary outcome to ensure that we have a sufficient number of cases to assess impact. Within the Pihlajalinna Työterveys cohort in 2015, there were 17,794 instances of sickness absences between 4 and 9 days in duration. This outcome is also supported by a large Finnish study on over 6000 municipal workers [26] which indicated that sickness absences of between 4 and 9 days were more prevalent (85.7 and 59.0 per 100 person-years for women and men, respectively) than those over 2 weeks (10+ days) in duration. The study also indicated that self-certified (1–3 days) absences were the most common (156.4 and 96.1 per 100 person-years for women and men, respectively), but these are not reliably available from the electronic patient register held by Pihlajalinna Työterveys. Longer sickness absences (between 2 weeks and 60 days) are less prevalent and had rates of 22.8 and 18.0 per 100 person-years for women and men, respectively, with long absences of over 60 days being relatively infrequent (4.7 and 4.4 per 100 person-years for women and men, respectively) [26]. Shorter (1–3 days) sickness absences were not included as a primary outcome, because they are not reliably available from the patient register, and they have been found not to be associated with disability pensions, except through their association with longer-term (3+ days) sickness absences [9]. Long (3+ days) sickness absence spells have been found to be predictive of work disability before the age of 55 [9] and regardless of health status [27].

#### Secondary outcomes

We will assess the following secondary outcomes:

1. Reduction in mean number of short term (1–3 days) sickness absences from the workplace per cluster from baseline after 1 year from the start of the intervention as measured by self-report recorded on OHS records or OHS records of sickness absence
2. Reduction of mean number of any form of work disability pensions as measured by an employee registering as receiving a work disability pension on the central pensions register from baseline to up to 2 years from the intervention as measured by the entry on the central pensions register
3. Reduction of mean number of long-term (9+ days) sickness absences from the workplace per cluster from baseline to 1 year after the intervention as measured by OHS records

### Power calculation

The pre-study power and sample size calculations for our main outcome were based on data collected in 2015 with estimates of the individuals between the ages of 18 and 65 from the 22 OH units (*n* = 24,892). The 22 clusters had an average size of 2300 individuals. We estimated the intra-cluster correlation coefficient to be 0.05. We used the n4means function in the CRTSize package in R [28], which takes into account that the outcome for individuals cannot be assumed to be independent. We used a two-sided alpha of 5% for our primary outcome, the sickness absences of medium (4–9) days, with an SD of 1.5. With 11 clusters randomised to each arm (assuming 2300 individuals per OHS), the study had 91% power to detect at least a 10% difference between individuals in the intervention and control OHS groups. These calculations showed that even with a 20% loss to follow-up or missing data, we would still have 90% power for the primary analysis.

### Data collection

Data on patient visits to the OHS unit were collected from a central patient register. Self-reported sickness absences of between 1-3 days are reported by the employer to the OHS unit. Pre-intervention data were extracted by the information technology service provider of Pihlajalinna Työterveys, who provided coded and anonymised patient data, a separate list of personal identifiers and a code for combining the patient data with the personal identifier. The list of personal identifiers was sent to the Finnish Centre for Pensions, which holds a registry of pensions, including disability pensions, for the entire population. The Finnish Centre for Pensions extracted data on pensions for the entire list of personal identifiers and coded it with the same code. The pseudonymised patient data and coded pension data were sent to the University of Tampere. The same process will be repeated for data collected during and after the intervention.

### Data management

The data from the Finnish Centre for Pensions and Pihlajalinna Työterveys were combined on the basis of patient codes at the University of Tampere using R software. Data were kept on a secure server, password-accessible only to the research team. Data accuracy will be checked by referring back to the OHS units for details where there are suspected errors in data entry. The Finnish Centre for Pensions register system checks the data automatically, and the data are routinely reviewed and corrected.

### Data analysis

Data will be analysed on the intention-to-treat principle. All employees registered at a cluster will be analysed as part of that cluster. We will use both R and IBM SPSS Statistics software (IBM, Armonk, NY, USA) to analyse the data. We will first examine the data using descriptive statistics. Between-group analysis will be conducted using Pearson’s chi-square test or Fisher’s exact test for categorical data and Student’s *t* test or the Mann-Whitney *U* test for continuous variables, depending on normality of a distribution. The normality of the distribution of the variable will be examined with the Kolmogorov-Smirnov test. The intervention effects for primary and secondary outcomes will be analysed using regression analysis, logistic regression will be used for dichotomous outcome variables, and Poisson regression will be used when the outcome variable is a count. In both analyses, we will examine the impact of, and control for, possible confounders. The regression analysis will result in ORs and rate ratios with 95% CIs. The results will be considered significant at *p* < 0.05. For analysing the differences in duration of sickness absences, we will use Kaplan-Meyer survival curves and the log-rank test.

### Data monitoring

Because the study did not include vulnerable populations, was considered not to represent a harm to participants, and was considered to have potential for routine practice, we deemed a data monitoring committee unnecessary.

### Ethical approval

Ethical approval was received from the Pirkanmaa Hospital District review board (Pirkanmaa Hospital District review board 10/03/2016, reference R16041).

## Discussion

The wealth of registers, especially in Nordic countries, allows for innovative research to be conducted with minimal need for additional data collection or trouble for the participants. Recent authors have argued that registry trials can have a wealth of benefits, but quality issues need to be taken into account [29]. Our pragmatic trial uses the combination of electronic health records with registries and tests a real-world intervention with a good study design and maximum external validity [30] with little disruption to the physicians implementing the study [31]. The trial is pragmatic in nature, and thus, while yielding results highly transferable to other settings, there are statistical challenges in design. These include, for example, the high variation in cluster size, which may reduce the power. Regardless of this, we expect the cluster and total sample sizes to be sufficient to detect a true difference, and the impact of the cluster size variation can be determined during analysis.

While interesting from a methodological standpoint, the practical impact of quality improvement in occupational health care has implications beyond this trial and this particular consortium. Through assessment of work-relatedness of each visit, primary care visits can be used effectively as a tool for secondary prevention and early intervention. These approaches can be used also in settings where occupational health care is not statutory. Studies have indicated that the recording of work-relatedness of visits is not optimal [32], and approaches such as this built into services could potentially improve reporting. Improved reporting and recognition can also contribute to collaboration between OHS and employers and general practitioners [33]. These issues are important particularly in Europe, where the population is ageing and life expectancy is increasing and where economic development depends on a healthy workforce.

Challenges to the success of the trial can be the scale of the intervention as well as follow-up time, which is short with respect to the outcome of disability pensions. Further funding will be sought to follow this cohort for a longer period. In addition, not all sickness absences can be extracted for all clients, because clients may be given sickness absence certificates from the public sector or specialised services. Changing behaviour on the basis of one training visit may be too optimistic; physicians may continue not to record work-relatedness or impact on work ability optimally; internal structures may not support collecting and following up employer visits; or employers may not be eager to invest in employee work ability. Through this randomised trial, we will be able to assess whether our approach works, and we will conduct important methodological work on using electronic registers for pragmatic trials. Through the planned process evaluation done alongside the trial, we will be able to assess what worked and what did not work, as well as how we should modify the intervention in future to ensure that supporting work ability is at the centre of primary care visits at occupational health care centres.

### Trial status

The trial began on 1 May 2016 and ended on 1 May 2017.

## Additional file

Additional file 1: SPIRIT checklist. (PDF 168 kb)

## Abbreviations

OH: Occupational health
OHS: Occupational health services
SPIRIT: Standard Protocol Items: Recommendations for Interventional Trials
